# The Role of the HMGB1–Pyroptosis Axis in Skeletal Muscle Dysfunction in COPD: Regulatory Mechanisms and Therapeutic Prospects

**DOI:** 10.3390/biology15211930

**Published:** 2026-10-24

**Authors:** Yangyang Cao, Qian Gao, Wendi Wang, Zhiwei Fan, Jun Wang, Weibing Wu

**Affiliations:** School of Exercise and Health, Shanghai University of Sport, Shanghai 200438, China; 2421517012@sus.edu.cn (Y.C.); gaoqian9608@126.com (Q.G.); 2511517004@sus.edu.cn (W.W.); zw1037228039@163.com (Z.F.); wangj0125@126.com (J.W.)

**Keywords:** chronic obstructive pulmonary disease, skeletal muscle dysfunction, high-mobility group box 1, pyroptosis

## Abstract

Skeletal muscle dysfunction is the most common extrapulmonary complication of chronic obstructive pulmonary disease (COPD) and has a significant impact on patients’ exercise capacity, quality of life, and survival. Although previous studies have elucidated some of the mechanisms underlying COPD-associated skeletal muscle dysfunction, the specific pathways through which COPD causes skeletal muscle injury remain to be fully explored. In patients with COPD, pathological factors such as long-term smoking, chronic inflammation, oxidative stress (OS), and pyroptosis can stimulate the release of high-mobility group box 1 (HMGB1), a classical damage-associated molecular pattern (DAMP), into the extracellular space. HMGB1 then binds to relevant receptors, triggers downstream responses, and activates pyroptosis. Characterized by inflammasome activation, membrane pore formation, and the release of pro-inflammatory mediators, pyroptosis is thought to play an important effector mechanism linking persistent inflammation to skeletal muscle injury. An in-depth analysis of the relationship between aberrant activation of the HMGB1–pyroptosis pathway and COPD-associated skeletal muscle dysfunction may help identify urgently needed new therapeutic strategies. Therefore, this article explores the molecular mechanisms of the HMGB1–pyroptosis pathway, summarizes current research on its role in COPD-associated skeletal muscle dysfunction, and discusses relevant intervention strategies, providing a theoretical basis for targeting this pathway to treat COPD-associated skeletal muscle dysfunction.

## Simple Summary

As a common extrapulmonary complication of chronic obstructive pulmonary disease (COPD), skeletal muscle dysfunction has a high incidence and can significantly impair patients’ exercise capacity, quality of life, and survival prognosis; however, its pathogenesis has not been fully elucidated. In the course of COPD, pathological stimuli such as smoking, chronic low-grade inflammation, and oxidative stress can promote the release of high-mobility group box 1 (HMGB1), a classic damage-associated molecular pattern, into the extracellular space; HMGB1 then binds to related receptors, activates downstream signaling, and induces pyroptosis. Because pyroptosis involves inflammasome activation, membrane pore formation, and the release of proinflammatory mediators, it is considered an important effector mechanism linking persistent inflammation to skeletal muscle injury. Therefore, the HMGB1–pyroptosis axis may not only be an important amplification mechanism of chronic inflammation, but also a key bridge linking pulmonary inflammation and skeletal muscle dysfunction. This article systematically summarizes the role and potential mechanisms of the HMGB1–pyroptosis axis in COPD-related skeletal muscle dysfunction and, based on the latest research advances in the structure and regulatory mechanisms of this axis, further discusses intervention approaches targeting this pathway and their application prospects in the treatment of COPD-related skeletal muscle dysfunction.

## 1. Introduction

Chronic obstructive pulmonary disease (COPD) is a common respiratory system disease marked by ongoing damage to the airways and alveoli. Its common symptoms include shortness of breath, coughing, sputum production, and fatigue [1]. COPD has become the third leading cause of death globally, imposing an ever-increasing medical and socioeconomic burden [2]. COPD-related injury can affect multiple organ systems throughout the body. Skeletal muscle dysfunction is a common complication in patients with COPD, with a reported prevalence of up to 63% [3]. Its primary clinical features include reduced skeletal muscle mass and volume, shifts in muscle fiber composition, and diminished muscle strength. These changes can significantly limit patients’ ability to perform daily activities and may contribute to poorer clinical outcomes [4]. Skeletal muscle strength and cross-sectional area—particularly those of the quadriceps—reflect patients’ exercise performance and prognosis more directly than body mass index and pulmonary function parameters [5]. Therefore, clarifying the molecular basis of COPD-associated skeletal muscle dysfunction is of great importance for further interpreting its clinical consequences and identifying possible treatment targets.

COPD-associated skeletal muscle damage is driven by a range of factors, including smoking exposure, chronic low-grade inflammation, hypoxia/hypercapnia, OS, mitochondrial dysfunction, malnutrition, and lowered physical activity [6]. However, how these factors interact to contribute to muscle atrophy and functional decline, as well as the key molecular events and regulatory networks involved, remain incompletely understood. High-mobility group box 1 (HMGB1) is an important damage-associated molecular pattern (DAMP) that has increasingly been recognized as a potential contributor to chronic inflammation in COPD and its extrapulmonary effects [7]. HMGB1 not only promotes the maintenance and amplification of airway inflammation but may also directly injure skeletal muscle tissue by activating the nuclear factor of kappa B (NF-κB) pathway [8,9]. Meanwhile, pyroptosis, a form of inflammatory programmed cell death, has attracted increasing attention for its role in chronic inflammatory diseases and skeletal muscle injury [10,11]. Notably, HMGB1 and pyroptosis may form a bidirectional inflammatory amplification loop. Specifically, HMGB1 can induce pyroptosis [12], whereas pyroptosis can directly cause skeletal muscle cell injury and amplify local and systemic inflammatory responses by releasing HMGB1 and other damage-associated molecular patterns (DAMPs). This two-way interaction may establish a vicious cycle that progressively exacerbates skeletal muscle atrophy and functional decline [13,14,15].

Studies have shown that HMGB1 levels are elevated in the peripheral blood of patients with COPD and are negatively correlated with thigh circumference and body mass index. Inhibition of HMGB1 expression or activity can, to some extent, ameliorate cigarette smoke extract (CSE)-induced pyroptosis and atrophy in myotubes, involving the HMGB1/toll-like receptor 4 (TLR4)-NLRP3-caspase-1-gasdermin D (GSDMD) signaling pathway [16]. These findings indicate that the HMGB1–pyroptosis axis may represent an important molecular link between COPD-associated inflammatory stimuli and skeletal muscle injury. However, direct evidence remains limited. The activation profile of this signaling axis in the skeletal muscle of patients with COPD, its upstream and downstream regulatory mechanisms, and its relative contribution compared with other pathogenic mechanisms remain to be clarified. Therefore, this article systematically summarizes the pathophysiological features of COPD-associated skeletal muscle dysfunction, the molecular basis of HMGB1 and pyroptosis, and their reciprocal regulation. Particular emphasis is placed on integrating available evidence indicating that COPD-related stimuli promote skeletal muscle atrophy and dysfunction via the HMGB1–pyroptosis signaling network. On this basis, the potential role of this pathway in disease onset and progression, as well as its prospects as a therapeutic target, is further analyzed.

## 2. Methods

In this review, we conducted a systematic and comprehensive literature search to investigate the potential association between the HMGB1–pyroptosis axis and skeletal muscle dysfunction in patients with COPD. The primary databases searched were PubMed, Web of Science, and Google Scholar, covering the period from September 2006 to September 2026. The search terms included “COPD”, “skeletal muscle dysfunction”, “muscle atrophy”, “muscle wasting”, “sarcopenia”, “HMGB1”, “pyroptosis” and “NLRP3 inflammasome”.

The eligibility criteria focused on the following aspects: studies involving patients with COPD or COPD-related cellular and animal models; studies addressing skeletal muscle dysfunction, muscle atrophy, or sarcopenia; studies investigating HMGB1, pyroptosis, or related signaling pathways; and studies classified as original research articles, systematic reviews, or relevant narrative reviews. Studies that examined HMGB1 or pyroptosis in isolation and had no direct relevance to COPD or skeletal muscle dysfunction were excluded. The included literature was subsequently synthesized to summarize the role, regulatory mechanisms, and potential research prospects of the HMGB1–pyroptosis axis in COPD-associated skeletal muscle dysfunction.

## 3. Pathophysiological Characteristics of Skeletal Muscle Dysfunction in COPD and the Molecular Basis of HMGB1 and Pyroptosis

### 3.1. Pathophysiological Features of COPD-Associated Skeletal Muscle Dysfunction

Skeletal muscle dysfunction in patients with COPD is primarily characterized by structural, metabolic, and functional alterations [17]. Structural changes in skeletal muscle include alterations in muscle composition, reductions in cross-sectional area, loss of muscle mass, and disruption of muscle protein homeostasis. Animal studies have shown that, after 16 weeks of CSE exposure, rats exhibited significant reductions in gastrocnemius muscle mass and cross-sectional area, along with a marked decline in muscle strength. These changes were accompanied by increased levels of the muscle atrophy-related factors muscle atrophy F-box protein 1 (Atrogin-1) and muscle-specific RING finger protein 1 (MuRF1) and a significant reduction in myosin heavy chain content [18]. Clinical studies have shown alterations in muscle composition in patients with COPD, characterized by a significant increase in the proportion of non-contractile tissues, such as adipose, fibrous tissues, and the extracellular matrix, and a marked decrease in voluntary muscle strength [19]. In addition, patients with COPD commonly exhibit reduced physical activity, skeletal muscle atrophy, decreased muscle cross-sectional area and volume, and diminished muscle strength, with the quadriceps being the most severely affected muscle group [20,21].

Metabolic disturbances in the skeletal muscles of patients with COPD are primarily associated with alterations in muscle fiber type. In animal studies, six months of cigarette smoke (CS) exposure resulted in a considerable transformation of type IIA muscle fibers into type IIX/B fibers in the soleus muscle of mice [22]. Clinical studies have shown that muscle fiber types are altered in patients with COPD. These alterations may enhance the metabolic efficiency of the diaphragm while reducing that of the lower-limb muscles [23]. In peripheral muscles, patients with COPD manifest substantial reductions in the proportions of type I muscle fibers, oxidative capacity, fiber size, and mitochondrial density, along with a marked increase in the proportion of type II muscle fibers. Collectively, these changes cause muscle atrophy and diminished muscle strength [24,25,26]. In addition, patients with severe COPD show decreased oxidase activity and impaired aerobic metabolic capacity in the quadriceps. In contrast, enzyme activity associated with anaerobic metabolism and anaerobic metabolic capacity are increased [27].

In COPD, skeletal muscle changes often include mitochondrial damage, problems with satellite cell function, and reduced muscle strength and endurance. A reduced number of mitochondria characterizes mitochondrial dysfunction, decreased oxidase activity and oxidative capacity, insufficient adenosine triphosphate (ATP) production, and increased reactive oxygen species (ROS) levels. These changes consequently reduced muscle strength and exercise capacity, and worsened muscle atrophy and fatigue [28]. Impaired satellite cell function is characterized by slowed autophagy and compromised muscle repair, resulting in reduced muscle fiber size [29]. In animal models of COPD, significant decreases in tibialis anterior muscle strength and grip strength have been observed [18,30]. Clinical studies have demonstrated that, compared with healthy individuals, patients with COPD have significantly reduced muscle strength, endurance, mass, and exercise capacity [31,32]. For example, both quadriceps muscle strength and endurance are markedly decreased in patients with COPD [26,32,33].

### 3.2. Molecular Basis of the HMGB1 and Pyroptosis

HMGB1 is a highly conserved nonhistone nuclear protein composed primarily of two tandem deoxyribonucleic acid (DNA)-binding domains, a C-terminal acidic tail, and an N-terminal region [34,35,36]. HMGB1 exists in four distinct redox isoforms: reduced (Re-HMGB1), disulfide (Ds-HMGB1), oxidized (Ox-HMGB1), and dimerized (Di-HMGB1) [37]. Under physiological conditions, HMGB1 is predominantly localized in the nucleus, where it participates in DNA replication, DNA repair, and chromatin stabilization [37,38]. Under pathological conditions, however, various factors—including CS [39], OS [40], disruption of calcium homeostasis [41,42], inflammation [43,44,45,46,47], and cell death [48,49,50,51,52,53,54]—can promote the translocation of HMGB1 from the nucleus to the cytoplasm and its release into the extracellular space. HMGB1 can be released through active secretion by immune cells or passively from damaged cells. Once released into the extracellular space, HMGB1 mainly acts as a DAMP. It can bind to several molecules or receptors, including lipoteichoic acid, toll-like receptors (TLR2, TLR4, TLR5), lipopolysaccharide (LPS), and the receptor for advanced glycation end products (RAGE), thereby activating downstream signaling pathways such as NF-κB and regulating inflammatory responses and immune processes in the body [55,56,57,58,59,60,61].

Pyroptosis is a form of programmed cell death characterized by inflammation and mediated by members of the gasdermin family [62]. Based on the mechanisms of upstream activation and the key executioner molecules involved, pyroptosis can be classified into the canonical, noncanonical, caspase-3/8-mediated, and granzyme-mediated pathways [11,63]. In the canonical pathway, pattern recognition receptors sense pathogen-associated molecular patterns or DAMPs and trigger inflammasome activation—for example, NOD-like receptor family pyrin domain containing 3 (NLRP3) and NLR family CARD domain containing 4. These inflammasomes then recruit and activate pro-caspase-1. Activated caspase-1 cleaves GSDMD, and the resulting N-terminal fragment creates pores in the cell membrane. Caspase-1 also cleaves the precursors of interleukin-1 beta (IL-1β) and interleukin-18 (IL-18) into their mature proinflammatory forms, thereby initiating an inflammatory response [11,64,65,66]. In the noncanonical pathway, bacteria and viruses directly activate human caspase-4/5 or murine caspase-11, which cleaves GSDMD, thereby inducing cell lysis [67]. In the caspase-3/8-mediated pathways, chemotherapy drugs or tumor necrosis factor (TNF) signaling activate caspase-3, which cleaves gasdermin E (GSDME). Under hypoxic conditions, programmed death ligand 1 translocates to the nucleus and upregulates gasdermin C expression, which is subsequently cleaved by caspase-8, ultimately leading to pyroptosis [68,69,70]. In the granzyme-mediated pathways, chimeric antigen receptor T cells release granzyme B, which cleaves GSDME, whereas CD8+ T lymphocytes and natural killer cells release granzyme A, which cleaves gasdermin B, thereby inducing pyroptosis [71,72,73]. Pyroptosis is characterized by plasma membrane rupture, DNA fragmentation, pore formation, cellular swelling, and cell lysis. During this process, inflammatory mediators and DAMPs, including HMGB1, are released [13]. Under physiological conditions, moderate pyroptosis contributes to host defense against pathogen invasion. However, excessive pyroptotic activation can provoke a vigorous inflammatory response, leading to extensive cell death and tissue damage and consequently causing the development of several inflammatory and autoimmune diseases [63].

## 4. Regulatory Role of the HMGB1–Pyroptosis Axis in COPD-Associated Skeletal Muscle Dysfunction

### 4.1. Aberrant Expression and Release of HMGB1 in COPD

In COPD, smoking, OS, air pollution, inflammation, and cell death can all induce the aberrant expression and release of HMGB1. Smoking is the primary etiological factor for COPD and an important upstream stimulus causing skeletal muscle dysfunction [74]. CS contains abundant oxidizing and proinflammatory components that can induce excessive production of ROS in lung tissue, disrupt the epithelial barrier, and promote the release of DAMPs and proinflammatory cytokines, ultimately resulting in persistent pulmonary inflammation [39]. Excessive ROS levels continuously increase. ROS can induce oxidative modification of HMGB1, leading to the formation of ds-HMGB1, which can simultaneously activate TLR4 and RAGE, thus causing the release of pro-inflammatory mediators [37,75]. Meanwhile, thioredoxin can reverse this oxidized state, reducing HMGB1 to the chemotactic form, re-HMGB1 [37,76]. In addition, HMGB1 can further form the pro-inflammatory di-HMGB1, which preferentially activates TLR4 on immune cells [37,58]. Prolonged and excessive ROS exposure can cause overoxidation of HMGB1, generating oxidized HMGB1 (ox-HMGB1), consequently promoting immune tolerance [37,77]. Intracellularly, low levels of ROS promote the formation of ds-HMGB1, whereas excessive ROS induce the generation of di-HMGB1, consequently enhancing cell viability. Extracellularly, both the reduced and oxidized forms of HMGB1 are able to coexist [37]. However, there is currently no direct evidence identifying which redox form of HMGB1 is responsible for skeletal muscle dysfunction in COPD.

In vitro studies have shown that CS can induce neutrophil necrosis, resulting in the extensive extracellular release of HMGB1 and other DAMPs, thereby further amplifying proinflammatory signaling in the airway epithelium [78]. In macrophages, CSE promotes the release of HMGB1 in both soluble and microvesicle-associated forms, with the nuclear export receptor chromosome region maintenance 1 playing an important role in this process [79]. CS can also promote the expression, translocation, and release of HMGB1 in pulmonary macrophages via nicotinic acetylcholine receptors, thereby inducing macrophage autophagy and migration and activating the NF-κB signaling pathway [80]. Animal studies have demonstrated that CS exposure induces the translocation of HMGB1 from the nucleus to the cytoplasm in mouse pulmonary epithelial cells, followed by its release into the extracellular space of lung tissue [81]. In mice exposed continuously to CS for 60 days, alveolar macrophage and neutrophil infiltration in bronchoalveolar lavage fluid (BALF) increased, while static lung compliance and the expression of matrix metalloproteinase-12 and HMGB1 were significantly elevated [82]. CSE can upregulate HMGB1 expression and promote its aberrant release by enhancing neutrophil autophagy, increasing neutrophil ROS production, and causing excessive ROS accumulation in skeletal muscle [83,84].

Compared with healthy individuals, neutrophils, alveolar macrophages, and bronchial epithelial cells from patients with COPD release HMGB1 into the airways. In addition, HMGB1 levels are significantly elevated in the lung tissue, sputum, BALF, and plasma of patients with COPD. These levels are positively correlated with disease severity but negatively correlated with the forced expiratory volume in one second (FEV1) [16,80,85]. Compared with patients with asthma, those with COPD exhibit higher HMGB1 levels in sputum. Moreover, HMGB1 levels are significantly higher in patients with stage II or III COPD than in those with stage I disease [86]. Further comparisons between smoking and nonsmoking patients with COPD have shown that HMGB1 levels are significantly elevated in epithelial cells, cells in the submucosal region, alveolar macrophages, peripheral airways, blood, and BALF of smokers. Meanwhile, smokers exhibit a lower post-bronchodilator ratio of FEV1 to forced vital capacity. HMGB1 levels in BALF are negatively correlated with FEV1 and the diffusing capacity of the lung for carbon monoxide, but positively correlated with IL-1β, suggesting that HMGB1 expression may be closely associated with COPD severity [87,88,89].

In addition to smoking, long-term exposure to air pollutants in patients with COPD can also induce HMGB1 upregulation. In animal models, exposure to vehicle exhaust or particulate matter with an aerodynamic diameter of ≤2.5 μm (PM2.5) similarly increases the expression of HMGB1 and RAGE, suggesting that exogenous particulate matter may aggravate COPD-like pulmonary injury by activating HMGB1-related signaling pathways [90]. Sustained exposure to toxic particles can induce necroptosis in pulmonary epithelial cells in COPD mice, significantly increasing the rate of cell death, thereby promoting the extensive release of HMGB1 and other DAMPs, stimulating the production of proinflammatory cytokines, and triggering pulmonary inflammatory and immune responses [91]. Furthermore, during the course of COPD, various cell types—including airway epithelial cells, alveolar macrophages, neutrophils, T lymphocytes, and dendritic cells—may undergo cell-type-specific pyroptosis, resulting in cell rupture and the release of additional HMGB1 [92,93]. Studies have shown that ds-HMGB1 is the predominant form of HMGB1 released during pyroptosis [94,95]; however, experimental evidence remains lacking on whether pyroptosis in the course of COPD also predominantly releases ds-HMGB1.

Studies have shown that aberrantly elevated HMGB1 levels can promote muscle atrophy by exacerbating inflammatory responses and activating the ubiquitin–proteasome system and the autophagy–lysosome pathway, whereas inhibition of HMGB1 can partially attenuate muscle atrophy [96]. As a potent upstream regulator of Atrogin-1 expression, HMGB1 can promote the expression of the muscle atrophy markers Atrogin-1 and MuRF1 through the TLR4/NF-κB signaling pathway, leading to reductions in muscle fiber diameter and number and ultimately resulting in muscle atrophy. Conversely, HMGB1 inhibition can alleviate inflammation and ameliorate muscle atrophy [9,96,97]. HMGB1 can promote macrophage polarization toward the M1 phenotype, impair satellite cell activation and differentiation, and disrupt muscle fiber regeneration, therefore causing muscle atrophy. It can also activate NF-κB and chemokine-related signaling in macrophages, additionally worsening the inflammatory response in COPD [93,98]. In addition, aberrantly elevated HMGB1 may further intensify inflammation by activating the pyroptotic pathway, bringing about decreased muscle mass, reduced myofiber area, and increased fibrosis, ultimately resulting in muscle dysfunction [97]. A research team found that serum HMGB1 and IL-18 levels were higher in patients with COPD than in healthy individuals, and that serum HMGB1 levels were significantly negatively correlated with thigh circumference in both the COPD cachexia group and the non-cachexia group; the higher the serum HMGB1 level, the smaller the thigh circumference [16]. This suggests that HMGB1 may directly or indirectly lead to skeletal muscle atrophy by mediating systemic inflammation.

In summary, CS, the primary stimulus in COPD, can promote the expression, nuclear translocation, and release of HMGB1 in neutrophils, alveolar macrophages, and bronchial epithelial cells through mechanisms involving OS and inflammation. HMGB1 levels are markedly elevated in the airways, blood, and lung tissue of patients with COPD and are positively correlated with disease severity but negatively correlated with pulmonary function and thigh circumference. Given that skeletal muscle dysfunction is also closely associated with COPD severity [29], HMGB1 may participate in the development and progression of COPD-associated skeletal muscle dysfunction. However, its precise role and underlying mechanisms remain to be elucidated.

### 4.2. Activation of Pyroptosis in COPD

During the pathological progression of COPD, CS and air pollution can also activate pyroptosis. In vitro studies have shown that CS significantly upregulates the expression of sphingosine-1-phosphate receptor 2, NLRP3, apoptosis-associated speck-like protein containing (ASC), caspase-1, GSDMD, IL-1β, and IL-18 in human bronchial epithelial (HBE) cells. The resulting pyroptotic activation contributes to airway inflammation and injury [99]. CS can induce pyroptosis in HBE cells via the ROS/NLRP3/caspase-1 pathway, thereby triggering inflammatory responses [100]. CSE can also activate macrophages to secrete tumor necrosis factor-alpha (TNF-α). Through a paracrine mechanism, this cytokine acts on tumor necrosis factor receptor 1 (TNFR1) receptors on skeletal muscle cells, initiating the TNF-α/TNFR1-NLRP3-caspase-1-GSDMD signaling cascade, which induces pyroptosis and myotube atrophy [101]. In addition, cell and animal experiments have shown that CS can also mediate pyroptosis through the HMGB1/TLR4 pathway, exacerbating inflammatory responses and skeletal muscle atrophy [16]. In animal studies, prolonged CS exposure significantly upregulated transient receptor potential vanilloid 4 (TRPV4) expression in the airway epithelial cells of mice. TRPV4 aggravated mitochondrial injury in response to CSE and mediated pyroptosis of alveolar epithelial cells through the NLRP3/caspase-1/GSDMD pathway [102].

In addition to smoke exposure, triggering receptor expressed on myeloid cells 1 (TREM-1) can induce pyroptosis by activating the NLRP3 inflammasome pathway, thereby increasing macrophage infiltration and aggravating pulmonary inflammation and injury in COPD mice. Inhibition of TREM-1 can alleviate these pathological changes [103]. LPS induces the upregulation of the long noncoding ribonucleic acid (RNA) growth arrest-specific 5, which can promote pyroptosis in COPD by regulating the microRNA-223-3p/NLRP3 signaling axis [104]. In addition, wood-smoke-derived biomass fuel PM2.5 can markedly induce pyroptosis and inflammatory responses in HBE cells from patients with COPD through both the canonical NLRP3/caspase-1/IL-1β-dependent pathway and an ATP/P2Y purinoceptor-dependent mechanism [105].

The degree of pyroptosis activation varies considerably across the stages of COPD, with the most pronounced response occurring during acute exacerbations. In the lung tissue of patients with stable COPD, the messenger ribonucleic acid (mRNA) expression levels of NLRP3 and IL-1β are upregulated, whereas caspase-1 and ASC are predominantly present in their inactive forms. These findings suggest that the NLRP3 inflammasome is not fully activated and that the pyroptotic response is therefore relatively limited [106,107]. By contrast, in patients with acute exacerbations of COPD, the gene expression levels of IL-1β, NLRP3, and caspase-1 are significantly increased. The levels of caspase-1, oligomerized ASC, and related cytokines, including IL-1β and IL-18, are elevated in sputum, while the concentration of IL-1β in peripheral plasma is also markedly increased. These findings indicate substantial activation of the NLRP3 inflammasome, which further triggers inflammatory responses and pyroptosis [106,107,108]. As the disease progresses into the clinical recovery phase, the levels of caspase-1, oligomerized ASCs, and the related cytokines IL-1β and IL-18 in sputum gradually decrease, suggesting that the pyroptotic response is progressively attenuated [106].

Pyroptosis exerts context-dependent effects in muscle injury: in the early stage of injury, pyroptosis can prevent steatosis and promote muscle repair and regeneration; however, under severe pathological conditions, pyroptosis triggers intense inflammation, leading to myofiber destruction, fibroblast activation, and muscle fibrosis, ultimately resulting in loss of muscle function [109]. During inflammation and tissue injury in patients with COPD, macrophages become activated; however, owing to impaired phagocytic and exocytic functions, they are unable to promptly eliminate damaged and dead cells and cellular debris generated during the inflammatory process [110]. As a result, the cellular debris, inflammatory mediators, and DAMPs released during pyroptosis may not be effectively cleared. Their accumulation may further intensify the pyroptotic response, forming a vicious cycle that locally amplifies inflammation and worsens tissue injury. Thus, pyroptosis may contribute to the development of both COPD and its somatic muscle dysfunction, and further increase inflammation and tissue damage during disease exacerbation.

### 4.3. The HMGB1–Pyroptosis Axis Mediates Skeletal Muscle Injury in COPD

In COPD, there may be close functional coupling between HMGB1 and pyroptosis, forming an inflammation-positive feedback loop that mutually reinforces them. Under stimuli such as CS, OS, infection, hypoxia, and cell death, HMGB1 can be released through active secretion or passive leakage and accumulate in extracellular environments such as the blood and airways [8,111]. It then binds to the TLR4 and RAGE receptors on the cell membrane, activates the NF-κB signaling pathway, and induces the release of pro-inflammatory factors such as TNF-α, IL-1β, and interleukin-8, thereby triggering pulmonary airway inflammation [8,16,111,112].

TLR4 is an important pattern-recognition receptor in innate immune responses, and its activation can induce chronic inflammation, increase ROS production, and exacerbate OS. Studies have shown that both CS exposure and OS can upregulate TLR4 expression [112,113]. After disulfide HMGB1 binds to the TLR4/Myeloid differentiation protein-2 complex, it activates the myeloid differentiation factor 88-dependent signaling pathway, thereby promoting the nuclear trans-location of NF-κB and downstream inflammatory responses [37,113]. RAGE is highly expressed in lung tissue and is closely associated with the occurrence and progression of COPD [114]. After binding to RAGE, DAMPs such as HMGB1 can activate transcription factors including NF-κB, signal transducer and activator of transcription 3, and specificity protein 1, thereby inducing inflammatory responses and cell death [114,115]. In addition, positive feedback loops may form between RAGE and NF-κB, as well as TLR4 and OS, continuously amplifying inflammation [116,117].

Once activated, NF-κB can promote the upregulation of the transcription of inflammation-related genes such as NLRP3, pro-interleukin-1 beta (pro-IL-1β), pro-interleukin-18 (pro-IL-18), TNF-α, and interleukin-6 (IL-6), thereby providing the priming signal for the activation and assembly of the NLRP3 inflammasome [112,118,119]. COPD is often accompanied by persistent ROS accumulation, calcium homeostasis imbalance, and mitochondrial dysfunction, all of which can further promote NLRP3 inflammasome assembly [120,121,122,123]. NLRP3 may be an important hub linking the recognition of danger signals to skeletal muscle pyroptosis. In relevant models, the expression of NLRP3, GSDMD, caspase-1, ASC, IL-1β, IL-18, and the atrophy-related molecules MuRF1 and Atrogin-1 is increased in the gastrocnemius muscle, whereas these changes are significantly attenuated after NLRP3 knockout [124]. In an in vitro myotube model, treatment with CSE or recombinant HMGB1 can induce increased expression of NLRP3 inflammasome components and pyroptosis markers, accompanied by myotube atrophy; these effects are markedly reduced after NLRP3 knockout [16].

**Figure 1:**
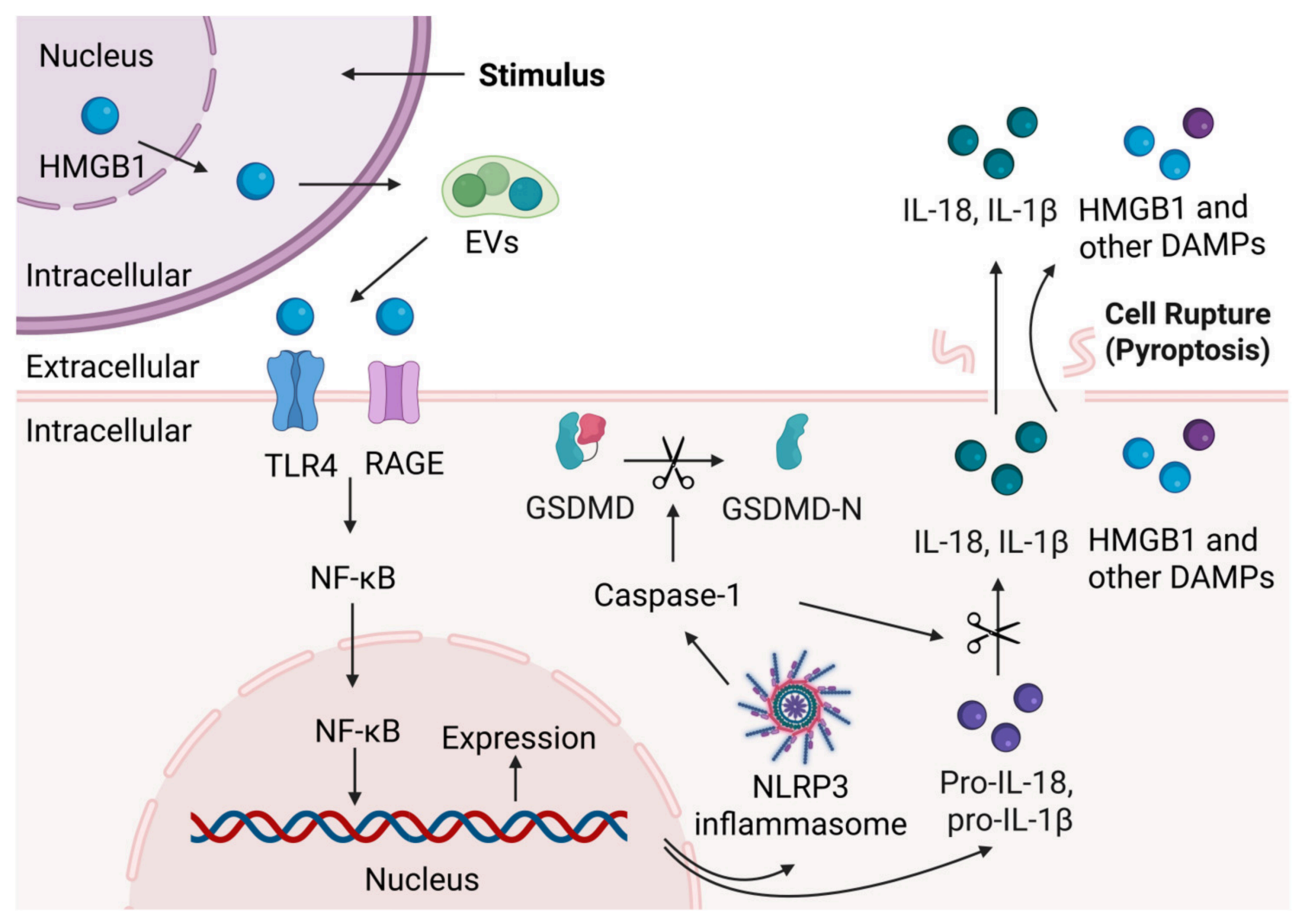
This is a mechanism of the HMGB1–pyroptosis axis. Following stimulation, HMGB1 moves from the nucleus into the extracellular space, and then it binds to TLR4 or RAGE on the cell surface. This activates NF-κB signaling and increases the expression of NLRP3, pro-IL-1β, and pro-IL-18. The NLRP3 inflammasome then activates caspase-1, which cleaves GSDMD, forming pores in the plasma membrane. Caspase-1 also processes pro-IL-1β and pro-IL-18 into their mature forms, IL-1β and IL-18, which are released from the cell. HMGB1 and other DAMPs released during cell rupture can further intensify inflammation and tissue damage. Abbreviations: HMGB1, high-mobility group box 1; EVs, extracellular vesicles; TLR4, toll-like receptor 4; RAGE, receptor for advanced glycation end products; NF-κB, nuclear factor of kappa B; GSDMD, gasdermin D; GSDMD-N, gasdermin D N-terminal fragment; NLRP3, NOD-like receptor family pyrin domain containing 3, Pro-IL-18, pro-interleukin-18; Pro-IL-1β, pro-interleukin-1 beta; IL-18, interleukin-18; IL-1β, interleukin-1 beta; DAMPs, damage-associated molecular patterns.

After assembly of the NLRP3 inflammasome, ASC and caspase-1 are recruited to form a multimolecular complex, thereby activating caspase-1 [125]. In addition to promoting the maturation of pro-IL-1β and pro-IL-18 and the secretion of their mature forms, activated caspase-1 cleaves GSDMD, enabling pore formation in the cell membrane and inducing pyroptosis [126]. Pyroptotic cells can release HMGB1, lactate dehydrogenase, ASC specks, and other DAMPs/injury markers [15,127]. IL-1β released from pyroptotic cells can act on neighboring cells or immune cells, further enhancing inflammasome activity [128]. Released DAMPs can recruit neutrophils and macrophages, promote the secretion of pro-inflammatory factors and secondary pyroptosis, and thereby exacerbate tissue damage [129]. Released HMGB1 can, in turn, act as an upstream danger signal and continuously promote the expression of NLRP3 and inflammasome precursor molecules through the TLR4/RAGE–NF-κB pathway, establishes a positive-feedback signaling axis involving HMGB1 release, activation of the pyroptosis pathway, and amplification of inflammation [13,14,15,130]. The mechanism of the HMGB1–pyroptosis axis is illustrated in Figure 1.

Under the context of COPD, multiple cell types can produce extracellular vesicles (EVs). As important carriers of intercellular signaling, EVs are widely distributed in body fluids such as blood, BALF, and sputum. They can transport proteins, lipids, and RNA, thereby participating in the propagation of inflammation [93,131]. EVs derived from different sources play different roles in the development and progression of COPD [131]. EVs originating from lung cells, such as macrophages, neutrophils, epithelial cells, and endothelial cells, may trigger systemic inflammation, which may impair muscle anabolic and restorative capacity while increasing catabolism [74,93]. Animal studies have confirmed that EVs enriched in pro-inflammatory factors such as IL-6 can induce muscle atrophy by altering mitochondrial metabolism and myogenesis [132]. Cell-based and animal experiments have also demonstrated that exosomes containing proline/arginine-rich end and leucine-rich protein, released by lung epithelial cells during CS exposure, can circulate to skeletal muscle, accelerate histone deacetylase 2 degradation, and induce skeletal muscle senescence and atrophy [133]. These data indicate that EVs act as key messengers in the COPD lung–muscle axis. Several studies in cachexia cell and animal models have shown that HMGB1 contained in mouse CT26 colon cancer cell-derived exosomes can activate the TLR4/NF-κB signaling pathway, resulting in reduced myotube diameter and increased expression of muscle atrophy-related proteins, including Atrogin-1 and MuRF1. In addition, it has been reported that LPS can induce hepatic macrophages to release HMGB1-containing extracellular EVs, in which HMGB1 triggers pyroptosis in hepatocytes by activating the NLRP3-caspase-1 pathway in target cells (hepatocytes) [134]. These results indicate that, during the progression of COPD, HMGB1-containing exosomes may contribute to skeletal muscle injury and promote muscle atrophy. However, whether these exosomes originate from the lungs, skeletal muscle, or both remains to be further clarified experimentally. Moreover, because serum HMGB1 levels are significantly higher in patients with COPD than in healthy controls [16], it also remains to be determined whether circulating HMGB1 can be directly transported to myocytes and induce pyroptosis.

Currently, only cell and animal models of COPD have confirmed that the HMGB1/TLR4 signaling pathway can induce skeletal muscle pyroptosis [16]. In addition, related cell and animal models have shown that the HMGB1/RAGE signaling pathway can promote pyroptosis in airway smooth muscle cells in childhood asthma [135]. To summarize, in COPD-related skeletal muscle injury, HMGB1 may act upstream of CSE-induced skeletal muscle pyroptosis, promoting NLRP3 inflammasome activation through the TLR4/RAGE–NF-κB signaling pathway and inducing caspase-1 maturation and GSDMD cleavage. The contents released during pyroptosis contain abundant HMGB1 [15], which may further activate the above pathway, continuously amplifying local inflammation and aggravating skeletal muscle injury. Therefore, the HMGB1–pyroptosis axis may not only serve as an important amplification mechanism in chronic inflammation but also as a key mediator of abnormal communication along the lung–skeletal muscle axis. However, experimental evidence for HMGB1/RAGE signaling-mediated skeletal muscle pyroptosis in COPD is still lacking, and no clinical evidence of pyroptosis has yet been obtained in the skeletal muscle of patients with COPD.

**Figure 2:**
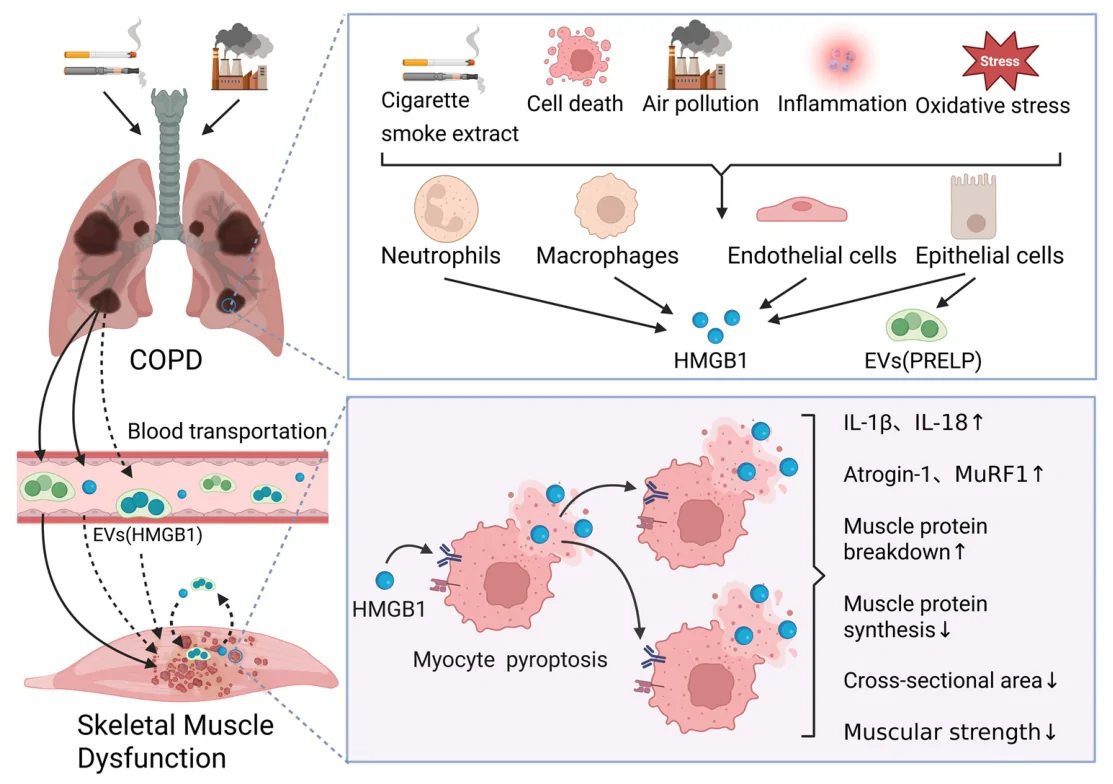
Schematic diagram of selected mechanisms involved in skeletal muscle dysfunction in COPD. Exposure to factors such as CSE causes lung epithelial cells to release extracellular vesicles (EVs) containing PRELP. These EVs enter the bloodstream and reach skeletal muscle, where they contribute to muscle dysfunction. Additionally, in COPD, agents such as CS, air pollution, inflammation, OS, and cell death stimulate neutrophils, macrophages, endothelial cells, and epithelial cells to release HMGB1 into circulation. HMGB1 can induce pyroptosis in muscle cells, leading to elevated levels of IL-1β and IL-18 and increased expression of atrophy-related proteins, including Atrogin-1 and MuRF1. This change pushes muscle protein balance toward breakdown. As a consequence, muscle fibers become smaller and weaker, resulting in diminished muscle function. Solid arrows show pathways that have been confirmed, while dashed arrows represent those that are still uncertain. In the figure, upward arrows mean an increase or activation, and downward arrows mean a decrease or suppression. Abbreviations: COPD, chronic obstructive pulmonary disease; EVs, extracellular vesicles; HMGB1, high-mobility group box 1; IL-1β, interleukin-1 beta; IL-18, interleukin-18; Atrogin-1, muscle atrophy F-box protein 1; MuRF1, muscle-specific RING finger protein 1; PRELP, proline/arginine-rich end and leucine-rich protein.

In addition to the HMGB1–pyroptosis axis and common contributors to skeletal muscle dysfunction in COPD, such as chronic inflammation, OS, nutritional imbalance, hypoxemia, and lower physical activity [6], studies have found that long-term CS exposure can upregulate the receptor activator of nuclear factor κB ligand (RANKL)/receptor activator of nuclear factor κB (RANK) pathway in the skeletal muscles of COPD mice, leading to muscle dysfunction and atrophy; inhibition of RANKL can improve these changes, suggesting that the RANKL/RANK pathway is also involved in the development and progression of COPD-related skeletal muscle dysfunction [30]. COPD leads to abnormal expression of mitochondria-related signaling molecules in skeletal muscle, including peroxisome proliferator-activated receptors and peroxisome proliferator-activated receptor gamma coactivator 1-alpha, resulting in reduced mitochondrial content and dysregulated mitochondrial biogenesis, therefore contributing to skeletal muscle dysfunction in COPD [28]. In patients with COPD, dysregulated expression of myokines such as IL-6, interleukin-15, myostatin, irisin, and insulin-like growth factor (IGF)-1 can weaken mitochondrial protective effects, disrupt the dynamic balance between skeletal muscle protein synthesis and degradation, and impair the regenerative and reparative capacity of skeletal muscle satellite cells, thereby exacerbating skeletal muscle atrophy [136]. Skeletal muscle injury in patients with COPD can promote satellite cell senescence, which in turn leads to autophagic dysfunction, resulting in reduced myofibrillar protein synthesis, increased protein oxidation, and decreased muscle fiber diameter, eventually accelerating muscle atrophy [29]. Overall, these factors may contribute to skeletal muscle dysfunction in COPD, and in some individuals, multiple factors may interact, with their relative contributions to the disease varying from person to person [6]. A schematic diagram of selected mechanisms involved in skeletal muscle dysfunction in COPD is shown in Figure 2.

## 5. Targeted Therapeutic Strategies for the HMGB1–Pyroptosis Axis

### 5.1. Therapeutic Strategies for Regulating HMGB1

Regulating HMGB1 may be a possible strategy for targeting the HMGB1–pyroptosis axis. Targeted therapy and exercise intervention against HMGB1 may help inhibit HMGB1 release and block its binding to related receptors [137,138], thereby suppressing pyroptosis and reducing the spread of inflammatory responses and muscle damage.

Extracellular HMGB1 is an important upstream danger signal that drives inflammasome activation and pyroptotic responses. By inhibiting HMGB1 release or neutralizing its extracellular activity, innate immune activation can be directly suppressed, therewith alleviating inflammation and tissue damage [139]. In a CS-induced mouse model of COPD, small interfering RNA (siRNA)-mediated downregulation of HMGB1 in lung tissue reduced the secretion levels of IL-6 and IL-1β, as well as the expression levels of RAGE and TLR4 and the phosphorylation level of NF-κB p65, compared with those in the COPD model group and the si-NC group, suggesting that HMGB1 downregulation may alleviate pulmonary inflammation and tissue injury through inhibition of NF-κB signaling [140]. In addition, microRNA-181a-5p can target HMGB1 and suppress the NF-κB pathway, thereby attenuating inflammatory responses and lung injury in COPD mice [141]. In an LPS-induced mouse model of acute lung injury, early administration of high-dose ethyl pyruvate markedly inhibited HMGB1 release, reduced inflammatory cytokine secretion, decreased the pulmonary permeability index, and significantly improved survival in mice [42,142]. Binding of HMGB1 to cluster of differentiation 24 (CD24) and sialic acid binding Ig-like lectin 10 (Siglec-10) blunts its activity, dampens NF-κB signaling, lowers inflammatory mediator production, and limits tissue damage [143]. Glycyrrhizic acid forms a stable complex with HMGB1, thereby interrupting the propagation of danger signals and easing inflammation [144]. Anti-HMGB1 antibodies can likewise neutralize HMGB1’s pro-inflammatory activity and biological effects, reducing inflammation and lung tissue injury [139]. As pulmonary inflammation resolves, lung cells secrete fewer HMGB1-containing exosomes, which helps restrain further spread of inflammation. In addition, studies have shown that glycyrrhizin, as an HMGB1 inhibitor, can directly bind HMGB1 and effectively ameliorate muscle atrophy and body weight loss by suppressing the NF-κB signaling pathway and downregulating the expression of the muscle atrophy-related genes Atrogin-1 and MuRF1 [97]. However, most strategies directly targeting HMGB1 are still supported primarily by preclinical evidence, and their target specificity, pharmacokinetic characteristics, and optimal timing of administration remain to be further clarified.

At the receptor level, another key and therapeutically tractable component of the HMGB1–pyroptosis axis is evident. HMGB1 primarily drives inflammatory signaling through TLR4 and RAGE, thereby promoting the priming and activation of the NLRP3 inflammasome [145]. Studies have shown that TLR4 inhibition can ameliorate muscle atrophy by blocking HMGB1 signaling and reducing downstream receptor activation [97]. HMGB1 antagonists, such as the peptide P5779, the m2G7, dexmedetomidine, and resveratrol, have been shown to effectively alleviate HMGB1/TLR4-mediated inflammatory disorders. For example, HMGB1 antagonists such as m2G7, recombinant HMGB1 Box A protein, and acetylcholine can inhibit HMGB1-mediated macrophage activation and endocytosis, thereby reducing inflammatory responses [146]. FPS-ZM1 can inhibit the HMGB1/RAGE signaling pathway, reduce ROS generation, apoptosis, and inflammation, thereby exerting protective effects on skeletal muscle [147,148]. Therefore, blocking the interaction between HMGB1 and its receptors may alleviate skeletal muscle atrophy and suppress the transcriptional priming of inflammatory mediators, thereby further reducing subsequent caspase-1/GSDMD-mediated pyroptosis. However, because these receptors are also involved in host defense against infection and tissue repair, long-term or systemic inhibition may carry risks, including immunosuppression and impaired tissue repair.

In addition to targeted and pharmacological therapies, appropriate exercise intensity and duration may reduce HMGB1 expression and attenuate inflammatory responses. Prolonged low-intensity treadmill training has been shown to inhibit HMGB1/TLR4/NF-κB signaling pathway expression in mice, reduce pulmonary inflammation, and markedly improve exercise capacity and respiratory function [149]. After a 4-week low-intensity aerobic exercise intervention, both HMGB1 mRNA expression and HMGB1 protein levels in mouse lung tissue were significantly reduced, accompanied by alleviation of pulmonary inflammation and edema, and improved survival [150]. In contrast, high-intensity exercise may markedly increase HMGB1 levels and induce acute tissue injury. High-intensity exercise can increase neutrophil counts and IL-1β, IL-6, TNF mRNA, and HMGB1 levels in athletes’ sputum, thereby exacerbating pulmonary inflammation [151]. High-intensity eccentric exercise can also cause skeletal muscle necrosis in mice and significantly increase HMGB1 levels, with a more pronounced effect observed 48 h after exercise [152].

Overall, HMGB1 appears to act upstream of danger-signal recognition, inflammasome activation, and pyroptosis, making it an encouraging therapeutic target in COPD. Blocking HMGB1 expression, release, or extracellular activity—or preventing binding to receptors such as TLR4 and RAGE—can dampen NF-κB/NLRP3 signaling, reduce inflammatory mediator release, and alleviate skeletal muscle atrophy and lung injury [137,138]. Moderate, low-intensity exercise may also help by downregulating the HMGB1/TLR4/NF-κB pathway, whereas high-intensity exercise may promote HMGB1 release and worsen pulmonary and skeletal muscle injury. Although targeting HMGB1 and its related signaling pathways offers a promising new strategy for the prevention and treatment of COPD, further studies are required to clarify its safety, optimal timing of intervention, and value for clinical translation.

### 5.2. Therapeutic Strategies for Regulating Pyroptosis

Directly blocking the execution phase of pyroptosis could limit HMGB1 release from dying cells and, at the same time, offer a downstream way to target the “HMGB1–pyroptosis” axis. So far, most pyroptosis-directed interventions in COPD have centered on the NLRP3 inflammasome. Once activated and assembled, NLRP3 drives caspase-1 activation, promotes the maturation and release of pro-IL-1β and pro-IL-18, and triggers pyroptosis. The NLRP3 inflammasome has therefore become a leading candidate target for pyroptosis-related treatment [153].

MCC950, a diarylsulfonylurea compound, is a potent and selective small-molecule inhibitor of NLRP3. Blocking ATP hydrolysis by NLRP3 prevents NLRP3 activation and inflammasome assembly, which, in mice, leads to reduced CS-induced pulmonary inflammation and lung injury [154]. Dimethyl fumarate suppresses NLRP3 inflammasome activation through the nuclear factor erythroid 2-related factor 2 (Nrf2)/heme oxygenase-1 (HO-1) pathway, reduces caspase-1 and GSDMD-N expression, and dampens pyroptotic responses, thereby lessening CS-induced inflammation and lung injury in COPD [155]. Schisandrin A has been used to treat various pulmonary diseases and promotes HO-1 expression via Nrf2, markedly reducing OS. At the same time, it inhibits the formation of the NLRP3/ASC/caspase-1 inflammasome complex, reduces IL-1β- and IL-18-mediated inflammatory responses, and GSDMD-induced pyroptosis, and consequently attenuates CS-induced lung injury in COPD mice [156].

In a CSE-induced rat model of COPD, the flavonoid compound (−)-epicatechin alleviated CSE-induced pyroptosis and pulmonary inflammation by enhancing Nrf2 activity, inhibiting NLRP3 inflammasome activation, reducing lactate dehydrogenase release and the number of caspase-1-positive cells, and lowering the secretion levels of IL-1β and IL-18 [157]. Propofol, a commonly used anesthetic agent, can upregulate Nrf2 expression, inhibit NLRP3 expression, and reduce lactate dehydrogenase release, thereby alleviating inflammation, apoptosis, pyroptosis, and OS, and exerting protective effects against CSE-induced injury in HBE cells [158]. Trimetazidine, a drug that improves skeletal muscle dysfunction in humans and mice, has been shown in both in vivo and in vitro studies to alleviate muscle atrophy by inhibiting NLRP3/GSDMD-mediated pyroptosis [159]. In addition, studies have shown that bone morphogenetic protein-7 (BMP-7), a potential therapeutic agent for osteoporosis, can not only inhibit HMGB1 activity but also downregulate NLRP3 inflammasome expression, thereby effectively suppressing pyroptosis and improving skeletal muscle dysfunction [118]. However, its role in COPD-related muscle atrophy remains to be further validated.

Beyond targeted regulation of the NLRP3 inflammasome, other approaches have also been reported to alleviate COPD-related pyroptosis. One study found that hydrogen sulfide can attenuate CS-induced pyroptosis by modulating the TLR4/NF-κB signaling pathway, improve cell viability, and thereby exert protective effects against the onset and progression of COPD [160]. Exosomes derived from human adipose-derived stem cells can effectively reduce CSE-induced airway mucus hypersecretion, pulmonary inflammation, and lung tissue injury by inhibiting pyroptosis in alveolar macrophages in mice [161].

In addition to pharmacological treatment, physical therapies may also ameliorate COPD-related inflammation and lung tissue injury by suppressing pyroptosis. Studies have shown that balneotherapy can reduce the production of serum inflammatory cytokines in COPD rats by downregulating the NLRP3/ASC/caspase-1 and TLR4/NF-κB/GSDMD signaling pathways, decrease the accumulation of CD4+ and CD8+ T cells in lung tissue, and alleviate pulmonary OS and inflammatory cell infiltration, thereby improving airway wall thickening, airway remodeling, and histopathological lung injury [162].

In summary, targeting the execution phase of pyroptosis may help reduce inflammatory cell death and HMGB1 release. Although the above interventions can, to some extent, inhibit pyroptosis and ameliorate COPD-related lung injury and skeletal muscle damage, their efficacy and safety still require further validation through additional basic research and high-quality clinical trials before clinical translation can be advanced. Moreover, this strategy may not fully block non-pyroptotic effects of HMGB1, such as chemotaxis, endothelial activation, and tissue remodeling. Therefore, in diseases characterized predominantly by persistent sterile inflammation, combined blockade of HMGB1 signaling and pyroptotic execution may offer superior therapeutic potential.

Overall, the HMGB1–pyroptosis axis may link several key pathological events, including the release of DAMPs, activation of innate immunity, inflammatory cell death, and skeletal muscle injury. Accordingly, interventional strategies targeting this axis include inhibition of HMGB1 release and extracellular activity [38], blockade of receptor-mediated inflammatory signaling [146], and direct suppression of the NLRP3 inflammasome [15], among others. Table 1 summarizes representative therapeutic strategies targeting the HMGB1–pyroptosis axis.

In addition to targeting the HMGB1–pyroptosis axis, other therapeutic approaches can also alleviate inflammation and skeletal muscle dysfunction in COPD. N-acetylcysteine, which has antioxidant, anti-inflammatory, antibiofilm-forming, and immunomodulatory properties, has been proven to be an effective adjunctive treatment for COPD [163]. Studies have found a negative correlation between the dietary inflammatory index and the incidence of COPD-associated sarcopenia [164]. Association of dietary inflammatory indices with sarcopenia and all-cause mortality in COPD patients; however, whether a low dietary inflammatory index diet can effectively reduce the occurrence of COPD-associated sarcopenia remains to be further validated. Exercise is a core component of physical therapy to address skeletal muscle dysfunction in patients with COPD, as it can alleviate mitochondrial dysfunction by improving mitochondrial phenotype and redox status, consequently enhancing skeletal muscle structure and function [28]. In addition, exercise can improve muscle strength, muscular endurance, and exercise capacity in patients with COPD by regulating myokine expression, hence improving prognosis [136]. Given patient variability, treatment plans should be developed via comprehensive assessment and individualized therapy to better ease symptoms.

## 6. Discussion

Taken together, the role of the HMGB1–pyroptosis axis in skeletal muscle dysfunction in patients with COPD remains poorly defined. Current evidence comes largely from cell and animal models exposed to CSE, with only limited direct evidence from skeletal muscle tissue of COPD patients. How the axis changes dynamically across disease stages, during acute exacerbations, and among different skeletal muscle groups—and how these changes relate to declining muscle function—is still unclear. In addition, the redox state and subcellular localization of HMGB1, its receptor preferences, and the specific cell types undergoing pyroptosis in skeletal muscle remain to be clarified. The interaction among pyroptosis, apoptosis, necroptosis, impaired autophagy, mitochondrial dysfunction, and disturbed protein metabolism also warrants further investigation.

Subsequent studies should better integrate clinical samples with animal and cell models, and combine longitudinal follow-up with multi-omics analyses to clarify the main sources of HMGB1 in COPD, its key target cells, its spatiotemporal dynamics, and the causal relationship between these features and the decline in skeletal muscle function. It will also be important to further dissect the molecular links between the HMGB1–pyroptosis axis and OS, mitochondrial injury, metabolic reprogramming, imbalanced muscle protein synthesis/degradation, dietary suppression, and exercise rehabilitation. At the translational level, prospective strategies for improving skeletal muscle dysfunction in COPD include targeting HMGB1 release and extracellular activity, blocking HMGB1–TLR4/RAGE interactions, inhibiting NLRP3 inflammasome activation, or interfering with caspase-1/GSDMD-mediated pyroptotic execution. However, given that HMGB1 and pyroptosis also play important physiological roles in host defense, immune regulation, and tissue repair, future efforts must pay close attention to intervention timing, dosage, targeting specificity, and safety, and should explore precision treatment approaches based on disease stage and patient stratification.

## 7. Conclusions

Overall, HMGB1-driven pyroptosis may offer a novel mechanistic perspective on skeletal muscle dysfunction in COPD. The HMGB1–pyroptosis axis may not only contribute to the persistent amplification of chronic inflammation in COPD but also serve as an important mediator of abnormal communication along the lung–skeletal muscle axis. Further elucidation of the role of this axis and its therapeutic tractability is expected to deepen understanding of the underlying pathological mechanisms and provide a basis for identifying potential therapeutic targets.

## Figures and Tables

**Table 1:** Representative Therapeutic Strategies Targeting the HMGB1–Pyroptosis Axis.

COPD-Specific Evidence			
Intervention Target	Representative Agent/Strategy	Mechanism of Action	Experimental Evidence
HMGB1	siRNA	Inhibition of the NF-κB pathway	Attenuated pulmonary inflammation in COPD mice [140]
	microRNA-181a-5p	Inhibition of the NF-κB pathway	Reduced pulmonary inflammation and lung injury in COPD mice [141]
NF-κB	Hydrogen sulfide	Inhibition of the TLR4/NF-κB pathway	Attenuated pyroptosis in COPD [141]
NLRP3	MCC950	Inhibition of ATP hydrolysis within NLRP3	Reduced pyroptosis, inflammation, and lung injury in COPD mice [154]
	Dimethyl fumarate	Activation of the Nrf2/HO-1 pathway and inhibition of NLRP3 inflammasome activation	Reduced pyroptosis, inflammation, and lung injury in COPD [155]
	Schisandrin A	Activation of the Nrf2/HO-1 pathway and inhibition of NLRP3/ASC/caspase-1 inflammasome complex formation	Reduced pyroptosis, inflammation, and lung injury in COPD mice [156]
	(−)-Epicatechin	Enhancement of Nrf2 activity and inhibition of NLRP3 inflammasome activation	Reduced pyroptosis and pulmonary inflammation in COPD rats [157]
	Balneotherapy	Inhibition of the NLRP3/ASC/caspase-1 and TLR4/NF-κB/GSDMD signaling pathways	Reduced inflammation and lung injury in COPD rats [162]
**COPD indirect evidence**			
**Interven-tion target**	**Representative agent/strategy**	**Mechanism of action**	**Experimental evidence**
HMGB1	Ethyl pyruvate	Inhibition of HMGB1 release	Protective effects against pulmonary inflammation and in acute lung injury models [42,142]
	CD24/Siglec-10	Inhibition of the NF-κB pathway	Reduced inflammation and tissue injury [143]
	Glycyrrhizic acid	Binding to HMGB1 to form a stable complex	Attenuated inflammation [144]
	Anti-HMGB1 antibody	Inhibition of HMGB1 pro-inflammatory activity or reduction in its biological effects	Reduced inflammation and lung tissue injury [139]
	Glycyrrhizin	Direct binding to HMGB1	Alleviated muscle atrophy and body weight loss [139]
	BMP-7	Inhibition of HMGB1 activation	Reduced pyroptosis and improved skeletal muscle dysfunction [118]
	Prolonged low-intensity exercise	Suppression of the HMGB1/TLR4/NF-κB pathway and reduction in HMGB1 mRNA and protein levels	Reduced pulmonary inflammation and edema and improved respiratory and exercise capacity in mice [149,150]
HMGB1 receptor level	m2G7/peptide P5779/resveratrol	Inhibition of the HMGB1/TLR4 pathway	Alleviated inflammation and improved muscle atrophy [97,146]
	FPS-ZM1	Inhibition of the HMGB1/RAGE pathway	Reduced apoptosis, suppressed inflammatory responses, and ameliorated skeletal muscle disease [147,148]
NLRP3	Propofol	Enhancement of Nrf2 activity and inhibition of NLRP3 inflammasome activation	Reduced pyroptosis, inflammation, and injury in HBE cells [158]
	Trimetazidine	Inhibition of the NLRP3/GSDMD pathway	Reduced pyroptosis and skeletal muscle injury [159]

Note: Based on whether the experimental evidence explicitly involves a COPD model, the relevant evidence is divided into “COPD-specific evidence” and “COPD indirect evidence.” This original table does not provide evidence of clinical translation; therefore, no “clinical translation status” category is included. Abbreviations: siRNA, small interfering RNA; NF-κB, nuclear factor of kappa B; COPD, chronic obstructive pulmonary disease; HMGB1, high-mobility group box 1; ATP, adenosine triphosphate; CD24, cluster of differentiation 24; Siglec-10, sialic acid binding Ig-like lectin 10; BMP-7, bone morphogenetic protein-7; mRNA, messenger ribonucleic acid; TLR4, toll-like receptor 4; RAGE, receptor for advanced glycation end products; NLRP3, NOD-like receptor family pyrin domain containing 3; Nrf2, nuclear factor erythroid 2-related factor 2; HO-1, heme oxygenase-1; ASC, apoptosis-associated speck-like protein containing CARD; GSDMD, gasdermin D; HBE, human bronchial epithelial.

## Data Availability

No new data were created or analyzed in this study. Data sharing is not applicable to this article.

## Abbreviations

The following abbreviations are used in this manuscript:

ASC	Apoptosis-associated speck-like protein containing
ATP	Adenosine triphosphate
Atrogin-1	Muscle atrophy F-box protein 1
BALF	Bronchoalveolar lavage fluid
BMP-7	Bone morphogenetic protein-7
CD24	Cluster of Differentiation 24
COPD	Chronic obstructive pulmonary disease
CS	Cigarette smoke
CSE	Cigarette smoke extract
DAMP	Damage-associated molecular pattern
DAMPs	Damage-associated molecular patterns
DNA	Deoxyribonucleic acid
EVs	Extracellular vesicles
FEV1	Forced expiratory volume in one second
GSDMD	Gasdermin D
GSDME	Gasdermin E
HBE	Human bronchial epithelial
HMGB1	High-mobility group box 1
HO-1	Heme oxygenase-1
IL-1β	Interleukin-1 beta
IL-6	Interleukin-6
IL-18	Interleukin-18
LPS	Lipopolysaccharide
mRNA	Messenger ribonucleic acid
MuRF1	Muscle-specific RING finger protein 1
NF-κB	Nuclear factor of kappa B
NLRP3	NOD-like receptor family pyrin domain containing 3
Nrf2	Nuclear factor erythroid 2-related factor 2
OS	Oxidative stress
Pro-IL-1β	Pro-interleukin-1 beta
Pro-IL-18	Pro-interleukin-18
RAGE	Receptor for advanced glycation end products
RANK	Receptor activator of nuclear factor κB
RANKL	Receptor activator of nuclear factor κB ligand
RNA	Ribonucleic acid
ROS	Reactive oxygen species
Siglec-10	Sialic acid binding Ig-like lectin 10
siRNA	Small interfering RNA
TLR4	Toll-like receptor 4
TNF	Tumor necrosis factor
TNF-α	Tumor necrosis factor-alpha
TNFR1	Tumor necrosis factor receptor 1
TREM-1	Triggering receptor expressed on myeloid cells 1
TRPV4	Transient receptor potential vanilloid 4
